# Drought and Shrub Encroachment Accelerate Peatland Carbon Loss Under Climate Warming

**DOI:** 10.3390/plants14152387

**Published:** 2025-08-02

**Authors:** Fan Lu, Boli Yi, Jun-Xiao Ma, Si-Nan Wang, Yu-Jie Feng, Kai Qin, Qiansi Tu, Zhao-Jun Bu

**Affiliations:** 1 Jiangsu Key Laboratory of Coal-Based Greenhouse Gas Control and Utilization, School of Environment and Spatial Informatics, China University of Mining and Technology, Xuzhou 221116, China; luf785@cumt.edu.cn (F.L.); qinkai@cumt.edu.cn (K.Q.); 2 Jilin Provincial Key Laboratory for Wetland Ecological Processes and Environmental Change in the Changbai Mountains, Institute for Peat and Mire Research, Renmin 5268, Changchun 130024, China; ybl@imufe.edu.cn (B.Y.); majx029@nenu.edu.cn (J.-X.M.); wangsn915@nenu.edu.cn (S.-N.W.); fengyj258@nenu.edu.cn (Y.-J.F.); 3 School of Statistics and Mathematics, Inner Mongolia University of Finance and Economics, No. 185 Bei Erhuan Road, Hohhot 010051, China; 4 Key Laboratory of Geographical Processes and Ecological Security in Changbai Mountains, Ministry of Education, School of Geographical Sciences, Northeast Normal University, Renmin 5268, Changchun 130024, China; 5 School of Mechanical Engineering, Tongji University, Shanghai 201804, China; tuqiansi@tongji.edu.cn

**Keywords:** peat decomposition, temperature sensitivity, microbial activity, CO_2_, *Sphagnum*, vegetation changes

## Abstract

Peatlands store substantial amounts of carbon (C) in the form of peat, but are increasingly threatened by drought and shrub encroachment under climate warming. However, how peat decomposition and its temperature sensitivity (*Q_10_*) vary with depth and plant litter input under these stressors remains poorly understood. We incubated peat from two depths with different degrees of decomposition, either alone or incubated with *Sphagnum* divinum shoots or *Betula* ovalifolia leaves, under five temperature levels and two moisture conditions in growth chambers. We found that drought and *Betula* addition increased CO_2_ emissions in both peat layers, while *Sphagnum* affected only shallow peat. Deep peat alone or with *Betula* exhibited higher *Q_10_* than pure shallow peat. Drought increased the *Q_10_* of both depths’ peat, but this effect disappeared with fresh litter addition. The CO_2_ production rate showed a positive but marginal correlation with microbial biomass carbon, and it displayed a rather similar responsive trend to warming as the microbial metabolism quotient. These results indicate that both deep and dry peat are more sensitive to warming, highlighting the importance of keeping deep peat buried and waterlogged to conserve existing carbon storage. Additionally, they further emphasize the necessity of *Sphagnum* moss recovery following vascular plant encroachment in restoring carbon sink function in peatlands.

## 1. Introduction

Northern peatlands store over 500 Pg soil carbon (C) and play a crucial role in the global C cycle [1,2,3]. The large amount of soil C storage is attributed to both the cold and waterlogged environmental conditions that inhibit decomposers due to low temperature and reduced oxygen availability [3,4], and the ecological engineer *Sphagnum* which depresses other vascular plants and microorganisms through its acidity [5,6]. However, current and future climate warming is expected to increase the likelihood of drought and alter vegetation, thereby threatening the C sequestration function of peatlands [7,8,9,10].

Climate warming may increase the decomposition rate in natural ecosystems by enhancing microbial activity and enzymatic reactions [11]. In response to rising temperature, the decomposition rate of soil organic matter typically increases exponentially [12]. The temperature sensitivity (*Q*_10_), defined as the factor by which the decomposition rate increases for every 10 °C rise in temperature [13], is commonly used to represent the temperature sensitivity of organic matter decomposition. The sensitivity increases with the molecular complexity of the substrate, and is influenced by substrate concentrations and the affinities of the enzymes to these substrates [12,14].

Climate warming, whether acting alone or in combination with factors such as nitrogen (N) deposition [15] and drainage [10,16], is driving vegetation change in northern peatlands. In this context, shrub encroachment has been widely observed in peatlands across North America [15,17], Europe [18,19], and Asia [20]. *Sphagnum* mosses, with hyaline cells in leaves and capillary space among shoots, possess an outstanding water-holding capacity [4,21], which helps reduce water loss during drought. However, as the dominant plants change from *Sphagnum* mosses to vascular plants, peat drying may be further exacerbated [22]. Consequently, the indirect effect of climate warming—through changes in vegetation composition—may outweigh their direct effect on C balance in peatlands [19,23,24].

Litters from graminoids, forbs, and shrubs contain more N and a higher quality of C compared to *Sphagnum*, making them easier to decompose [20,25,26]. *Sphagnum* has lower levels of phenolics than vascular plants [27,28]. Phenol oxidase can partially oxidize phenolics into simple organic compounds to support microbial activity [21,29,30]. Furthermore, phenol oxidase activity is highly sensitive to lignin content; generally, higher lignin content in peat correlates with increased phenol oxidase activity [31]. According to the “enzyme latch” hypothesis, enhanced phenol oxidase activity is likely to decompose more phenolic compounds, stimulating the activity of hydrolytic enzymes, and thereby promoting peat decomposition [21].

Soil moisture content is a key factor shaping the composition and function of the soil microbial community [32] and can modify the effect of the litter substrate on *Q*_10_ [33]. Microorganisms require water to sustain their physiological activities, and soil moisture also indirectly affects substrate and oxygen availability [34,35,36]. Warming is intensifying drought frequency and severity in northern peatlands by altering precipitation patterns and increasing evapotranspiration [37]. Additionally, drainage can lower the water table [38], creating drought-like conditions that reduce soil moisture content. This increase in oxygen availability may stimulate phenol oxidase activity to degrade phenolic compounds, thereby relieving the inhibition of hydrolytic enzyme activities [21,39]. However, the response of *Q*_10_ in peat decomposition to the interaction between vegetation change and drought remains largely unknown.

Peat, composed of partially decomposed organic matter, accumulates layer by layer over the years [4]. The content of recalcitrant components, such as aromatic contents in peat, generally increases with depth due to the loss of labile components, like carbohydrates, through decomposition [40]. Carbon dioxide (CO_2_) production is relatively low in the deeper layers of peat, primarily due to the poor quality of C sources available for microorganisms [41,42]. The CO_2_ production rate during the decomposition of soil organic matter is mainly limited by the availability of C sources for microorganisms [41], which is more significant in deep stable soil organic matter [40]. This is because the stability of deep soil organic C is attributed to a lack of fresh C for soil microbes [43,44]. However, deep peat C is more sensitive to temperature change, exhibiting a high *Q*_10_ value [45,46,47,48], as described by the Arrhenius function [22]. For example, *Q*_10_ increases from 1.9 for shallow peat to 2.2 for deep peat [48]. The effect of peat depth or recalcitrance on Q_10_ can be influenced by moisture content [49,50], but findings are contradictory: Q_10_ may increase from 1.8 to 3.5 as moisture declines [50,51], or conversely decrease from 3.2 to 2.1 [49].

Currently, shrub encroachment results in increased input of fresh root litter into deep peat, while anthropogenic disturbances such as drainage, peat extraction, and trampling are exposing more deep peat to fresh leaf litter [10,52,53]. Deep soil organic matter is more susceptible to fresh organic matter input compared to unstable soil organic matter, due to the limited energy availability for microorganisms in stable organic matter [54]. Thus, fresh litter, particularly that from shrubs, may greatly enhance deep peat decomposition through its priming effect [26]. However, we still know rather little about how peatland vegetation succession and soil moisture changes affect the vulnerability of the deep stable C pool under global warming.

We conducted an indoor experiment by adding *Sphagnum* shoots and *Betula ovalifolia* leaf litters to two different depths (8–13 cm and 35–40 cm below the peatland surface, respectively) of peat and cultivated them at five different temperatures and two different moisture levels. We measured peat soil respiration and microbial biomass carbon (MBC), and subsequently calculated *Q*_10_. We tested the following hypotheses: (1) the addition of fresh litter, especially shrub litter, will accelerate peat decomposition, particularly in deep peat, by providing high-quality C sources with increased N content; (2) drought (reduced moisture content) will lead to a higher *Q*_10_ for peat decomposition due to increased oxygen availability for aerobic microorganisms, suggesting that vegetation degradation and drought may threaten peatland C pools under climate warming; and (3) deep peat will decompose more slowly but exhibit a higher *Q*_10_ than shallow peat, as microbial activity is likely more strongly C-limited in deep peat.

## 2. Results

### 2.1. Soil Physicochemical Properties of Different Depths of Peat

Overall, the soil physicochemical properties, except for MBC, varied between the two depths (Table 1). Total carbon (TC) content was higher in shallow peat (42.02 ± 0.54%) compared to that in deep peat (30.81 ± 1.89%), while total nitrogen (TN) content showed an opposite trend (0.91 ± 0.03 in shallow peat vs. 1.44 ± 0.08 in deep peat). Consequently, the C:N ratio was higher in shallow peat (46.22 ± 1.27 mg kg^−1^) than that in deep peat (21.78 ± 3.46). Furthermore, the dissolved organic carbon (DOC) concentration was greater in deep peat (108.53 ± 3.32 mg kg^−1^) than in shallow peat (176.94 ± 12.29). These results clearly indicate a great difference in decomposition degree between the two depths. However, the MBC concentration was similar across both depths (63.29 ± 13.85 in shallow peat vs. 63.53 ± 9.28 in deep peat).

### 2.2. Peat CO_2_ Production Rate and Its Temperature Sensitivity

**Figure 1:**
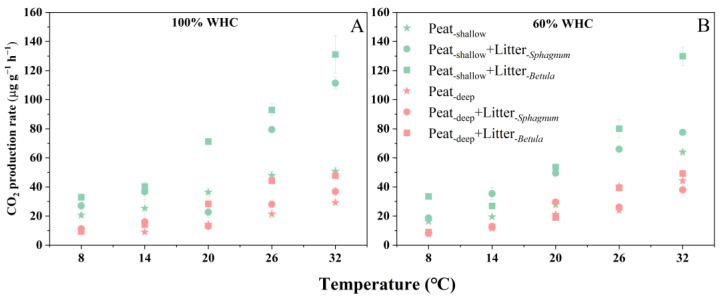
CO_2_ production rate of peats (μg g^−1^ h^−1^) at different depths in response to litter addition under different water-holding capacity (WHC) and temperature conditions: (**A**) 100% WHC data; (**B**) 60% WHC data. Values are mean ± SE (n = 4).

Depth, temperature, litter addition, and drought had a significant effect on the CO_2_ production rate, with notable interactions among these factors (Table 2). Overall, the CO_2_ production rate increased with temperature (Figure 1), and was higher in shallow peat than in deep peat (*p* < 0.001, Figure 1). The addition of both litters increased the CO_2_ production rate in the shallow peat, with *Betula* litter resulting in the highest CO_2_ production. In contrast, only the addition of *Betula* litter enhanced the CO_2_ production rate in deep peat (Figure 1). Figure S1 illustrated that the direct CO_2_ emissions from the added litter alone were minimal (0.08–0.51% of total CO_2_ production), suggesting that the observed increase in CO_2_ emissions was primarily due to stimulated peat decomposition. The effect of drought (60% WHC) on the CO_2_ production rate varied depending on the peat depth and temperature. In deep peat without litter addition, drought increased CO_2_ production, particularly under high temperature conditions (32 °C), where the rate under 60% WHC was ~15 μg g^−1^ h^−1^ higher than that under 100% WHC. In shallow peat without litter, the effect was less consistent, but under 32 °C, the rate under 60% WHC was still higher than that under 100% WHC, about 13 μg g^−1^ h^−1^ (Figure S2).

Without litter addition, the *Q*_10_ (temperature sensitivity) of shallow peat (1.47 ± 0.09 for 100% water-holding capacity (WHC) and 1.78 ± 0.03 for 60%) was lower than that of deep peat (1.76 ± 0.03 and 2.13 ± 0.11), with drought enlarging this difference (Table 3). The *Q*_10_ of the peat at 60% WHC was generally higher than that at 100% WHC for both depths. However, litter addition negated the effect of drought on *Q*_10_ (Table 3). Notably, deep peat with *Betula* litter addition exhibited the highest *Q*_10_ value (Table 3).

### 2.3. Microbial Metabolic Quotient

**Figure 2:**
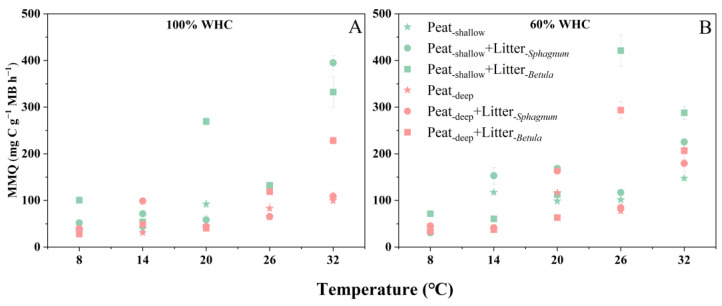
Microbial metabolic quotient (MMQ) (mg C g^−1^ MBC h^−1^) of peat at different depths in response to different litter additions under two water-holding capacity (WHC) conditions and temperature changes: (**A**) 100% WHC data; (**B**) 60% WHC data. Values are mean ± SE (n = 4).

Overall, the microbial metabolic quotient (MMQ) increased with temperature regardless of moisture content. Under the water-saturated condition, *Sphagnum* litter addition had a positive effect on the MMQ in shallow peat only at high temperatures (26 and 32 °C) (Figure 2A), but had a minor effect on the MMQ in deep peat at any cultivation temperature. In contrast, *Betula* litter addition influenced the MMQ in both shallow and deep peat. It increased the MMQ in shallow peat at nearly all cultivation temperatures except 14 °C, but in deep peat, it had an effect only at high temperatures.

Under the 60% WHC condition, the MMQ in shallow peat was higher than that in deep peat at relatively low cultivation temperatures (8 and 14 °C) (Figure 2B). However, this pattern changed with litter addition as the temperature increased. At 32 °C, the positive effect of *Betula* litter addition diminished compared to 26 °C, while it completely vanished in deep peat. Consistent with findings under 100% WHC, the *Sphagnum* litter addition had a minimal effect on the MMQ in deep peat at all temperatures, except 20 °C.

### 2.4. Relationships Between CO_2_ Production Rate and Microbial Biomass Carbon (MBC)

**Figure 3:**
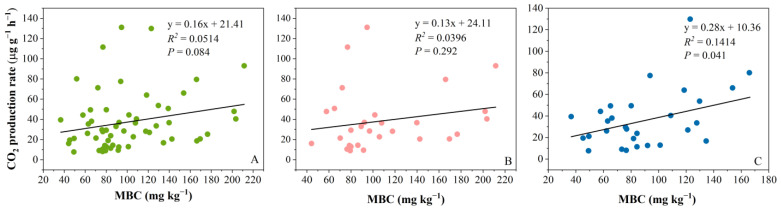
Relationships between the CO_2_ production rate (μg g^−1^ h^−1^) and microbial biomass carbon (MBC) (mg kg^−1^): (**A**) all data; (**B**) 100% WHC data; (**C**) 60% WHC data.

After cultivation, a difference in the distribution of MBC values between shallow and deep peat was observed. Approximately 50% of the MBC values in shallow peat exceeded 120 mg kg^−1^, whereas 100% of the MBC values in deep peat were less than 120 mg kg^−1^ (Figure S3). However, for all the data, the CO_2_ production rate exhibited a positive but marginal correlation with the MBC (*p* < 0.05). We found no correlation between the MBC and CO_2_ production rate for samples under 100% WHC (Figure 3B), but in the 60% WHC conditions (Figure 3C), MBC accounted for 14% of the variation in the CO_2_ production rate. No any other significant correlations between the CO_2_ production rate and MBC were found in other analyses according to peat depth and litter addition.

## 3. Discussions

### 3.1. Deep Peat Decomposes Slowly but Is More Sensitive to Warming

Our findings indicate that deep peat exhibits a slower CO_2_ production rate but is more sensitive to temperature change compared to shallow peat, aligning with our third hypothesis. An increase in Q_10_ with the peat decomposition degree has also been reported, indicating that carbon pools in deeper peat layers are particularly responsive to climate warming [48]. Given that deep soils store approximately three times more C than the top 20 cm of soils globally [43,55], even minor shifts in deep C cycling processes could strongly affect CO_2_ emission from peatlands. Thus, the effect of climate warming on the decomposition of deep organic matter warrants close attention.

Davidson and Janssens (2006) noted that the temperature sensitivity of organic matter decomposition rises with the complexity of substrate molecules [22]. In our study, the decreasing C:N ratio with depth suggests an increased decomposition degree, as microbes preferentially consume C-rich organic matter while recycling N. This results in higher relative N content in more decomposed soil [56,57,58]. The low C:N ratios observed in shallow (46) and deep (21) peat layers fall on the lower end of the typical range reported for ombrotrophic bogs, which is usually above 50 [58]. This pattern likely reflects historical disturbance at the study site, where intensive shallow drainage was implemented in the 1980s [59]. Such drainage can enhance aeration and promote microbial degradation of organic matter, thereby reducing the C:N ratio and increasing DOC concentrations, as documented in previous studies of disturbed peatlands [21,60]. Moreover, amino acids and other N-rich compounds with low C:N ratios have been shown to induce a strong priming effect on organic matter decomposition [61,62], providing a plausible mechanism for the higher temperature sensitivity of decomposition observed in deeper peat. The relatively low C:N ratio may also support the development of ‘wasteful’ microbial communities characterized by high extracellular enzymatic activity and greater carbon loss through respiration. In this study, as the temperature increased from 8 °C to 32 °C, MMQ values rose more in deep peat than in shallow peat, particularly at 60% WHC, indicating that microbial respiration in deep peat was more sensitive to temperature increases.

CO_2_ production during decomposition is primarily constrained by the availability of C sources for microorganisms [41,42]. Old organic matter remains stable partly due to a lack of fresh C resources for soil microorganisms [43,44]. In our study, the C:N ratio of deep peat was half that of shallow peat, and the TC content in deep peat was also much lower, indicating limited C availability for microorganisms and resulting in slower decomposition. Additionally, the difference in microbial biomass between shallow and deep peat may also contribute to the difference in decomposition, despite the absence of a significant relationship between the CO_2_ production rate and MBC under the 100% WHC condition (Figure S4).

### 3.2. Vegetation Succession Will Alter Peat Decomposition and Its Temperature Sensitivity (Q_10_)

Our study demonstrated that the addition of *Betula* litter increased the CO_2_ production rate in both peat layers and elevated the *Q*_10_ of deep peat, supporting our third hypothesis again. In contrast, *Sphagnum* litter addition enhanced CO_2_ production in shallow peat at higher temperatures, but had no effect on deep peat. Previous research indicates that shifts in vegetation composition under climate warming may lead to greater uptake of atmospheric CO_2_, potentially offsetting losses from ecosystem respiration [28,63]. However, our findings suggest that when global changes such as long-term warming lead to exposure of deep peat, shrub encroachment may release the old C from deep peat, triggering its mineralization. Similarly, Walker et al. (2016) also found that the presence of dwarf shrubs did stimulate the decomposition of ancient peat C, while *Sphagnum* did not [9]. Both the previous research and our study highlight the strong threat that shrub encroachment poses to the C pools in peatlands. Notably, CO_2_ production rates were greatest when *Betula* litter was added at temperatures exceeding 20 °C, further underscoring the profound effect of vascular plant expansion on peat decomposition amid climate warming [9].

Changes in plant litter can alter the supply of substrates necessary for microbial metabolism, thereby affecting the decomposition of soil organic matter [64]. *Betula* litter, rich in N, is readily decomposed by microorganisms [26,65]. The MMQ values for shallow and deep peat with *Betula* litter at 26 °C and 32 °C were all greater than those without, clearly demonstrating that high-quality litter enhances the metabolic rate of microorganisms at elevated temperatures. Interestingly, the MMQ peaked at 26 °C and then dropped sharply at 32 °C in 60% WHC peat with *Betula* litter addition, a trend not observed in 100% WHC peat. This suggests an optimal temperature range for microbial activity between 26 °C and 32 °C under both drought and shrub encroachment conditions. The CO_2_ production rate continued to trend upward from 26 °C to 32 °C, likely due to an increase in microbial biomass in response to the availability of new substrates [66].

The addition of *Sphagnum* litter had no impact on deep peat decomposition but enhanced the decomposition of shallow peat. This may be attributed to fresh *Sphagnum* litter providing a C source for microbes and enhancing their activity [67]. At higher temperatures (26 °C and 32 °C), the addition of *Sphagnum* litter increased the MMQ values of shallow peat by approximately 15.6% to 53.0% at 60% WHC, and 101.7% to 296.8% at 100% WHC. Additionally, litter application supplies not only C and energy but also other nutrients, such as N, for microorganisms [26]. In general, it is believed that the N-induced priming effect is stronger in old soil (with low N availability) and weaker in young soil (with high N availability) [26,68]. However, deep peat, characterized by poorer nutrient availability and more complex organic compounds, requires more nutrients and a higher activation energy to stimulate decomposition [69]. Compared to *Betula* litter, *Sphagnum* litter is nutrient-poor (Figure S5) but rich in organic acid [5], which may inhibit microbial activity [4,5]. Müller et al. (2023) also proved that *Sphagnum* litter suppresses extracellular enzyme activity [70]. It was observed that the addition of *Sphagnum* litter generally had little to no effect on the MMQ values of deep peat and even reduced the MMQ values by approximately 14.0% for 60% WHC deep peat at 32 °C and by 17.8% for 100% WHC deep peat at 26 °C, supporting this explanation.

Peatlands are currently experiencing degradation worldwide, driven by agriculture drainage, forestry, livestock overgrazing, peat mining, and pollution from human activity [3]. In such scenarios, dominant plants may shift from *Sphagnum* to vascular plants, accelerating peat respiration in both shallow and deep layers. Our study highlights that this shift may increase the vulnerability of long-stored peat carbon to decomposition under warming conditions. Although we did not assess aboveground plant productivity or the full ecosystem C balance, our findings suggest that restoring *Sphagnum*-dominated vegetation may help preserve the long-term stability of deep peat carbon stocks. This finding is crucial for guiding the restoration of vegetation and C-sink function in degraded peatlands.

### 3.3. Moisture Content Is Vital for Peat Decomposition and Its Temperature Sensitivity

Drought conditions significantly increased the *Q*_10_ value, which increased from 1.47 to 1.78 for shallow peat and from 1.76 to 2.13 for deep peat. This aligns with a previous study suggesting that high soil moisture content (flooded state) in a peatland reduces the temperature sensitivity of CO_2_ emission [50]. In perennially waterlogged peat, decreased water content enhances the high oxygen availability, which can increase the abundance of bacteria and fungi, as well as the activities of phenol oxidase and then hydrolytic enzymes, thereby accelerating aerobic respiration in soil microbial communities [21,34,39,71]. A significant positive relationship between the CO_2_ production rate and MBC was observed under the 60% WHC condition in this study. Furthermore, based on the MMQ results, a decrease in soil moisture content led to an increase in MMQ values, ranging from 0.6% to 152.7% in shallow peat and from 19.9% to 157.6% in deep peat. This indicates that the reduced water content significantly accelerated the aerobic respiration of microbial communities.

Our study revealed that the maximum Q_10_ value occurred in deep peat with 60% WHC when *Betula* litter was added, suggesting that water table drawdown and shrub encroachment could greatly accelerate C loss from peat under climate warming and threaten the stability of deep C pools. This phenomenon may result from two mechanisms: first, the enhanced oxygen availability at a lower moisture content favored aerobic microorganisms, increasing their abundance and activity; second, increased phenolics and lignin content from *Betula* litter addition may boost phenol oxidase activity, facilitating the decomposition of the organic matter rich in lignin and recalcitrant compounds in deep peat [72,73]. In deep peat at 60% WHC, the highest MMQ was observed with the addition of *Betula* litter at 26 °C and 32 °C.

## 4. Materials and Methods

### 4.1. Site Description

The Baijianghe peatland (42°9′36″ N–42°10′5″ N, 126°43′25″ E–126°44′19″ E) in the Changbai Mountains in northeastern China was selected for experimental material collection. The peatland is located at an elevation of 770–790 m and experiences a continental monsoon climate within a cold temperate region, with mean annual precipitation ranging from 775 to 930 mm and a mean annual air temperature of 3 °C [10]. Covering an area of approximately 2.45 km^2^, much of the peatland underwent intensive shallow drainage in the late 1980s [59]. The peat has an average pH of 5.8 and generally reaches a depth of about 1.5–3.0 m [74]. In undrained and near-pristine areas, *Sphagnum divinum* Flatberg & Hassel and S. *flexusoum* Dozy & Molk are dominant. In contrast, drained areas are dominated by the vascular plant *Potentilla fruticose* L., which covers nearly 100%, while *Sphagnum* mosses are rare, with less than 5% cover [59].

### 4.2. Soil and Litter Collection

In October 2021, four *Sphagnum divinum* hummocks, each with a diameter of 1–1.2 m and a water table depth of 20–25 cm, were selected in the peatland. Shallow peat (8–13 cm below the hummock surface), with a lower von Post decomposition degree H1, and deep peat (35–40 cm below the hummock surface), with a higher decomposition degree H3 were extracted from the four hummocks. A total of eight peat monoliths were collected from four microsites (2 depths × 4 replicates). After collection, visible plant residuals and gravels in each peat monolith were removed, and peats from the same depths at each microsite were mixed evenly. All peat materials were stored in an icebox and promptly transferred to the laboratory, where they were separated into two parts. One part was kept at 4 °C for microbial biomass carbon (MBC), dissolved organic carbon (DOC), total carbon (TC), and total nitrogen (TN) determination; the other part was used for the incubation experiment.

Fresh *Sphagnum divinum* shoots were collected from living moss plants, and the 2 cm portion below the capitulum—representing the current year’s growth—was clipped and used as *Sphagnum* litter. For *Betula ovalifolia*, naturally senesced leaves from the current year were collected from the ground surface. Both types of litter were cut into approximately 2 mm pieces, and then dried at 60 °C for 72 h for later use.

### 4.3. Incubation and CO_2_ Flux Measurement

**Figure 4:**
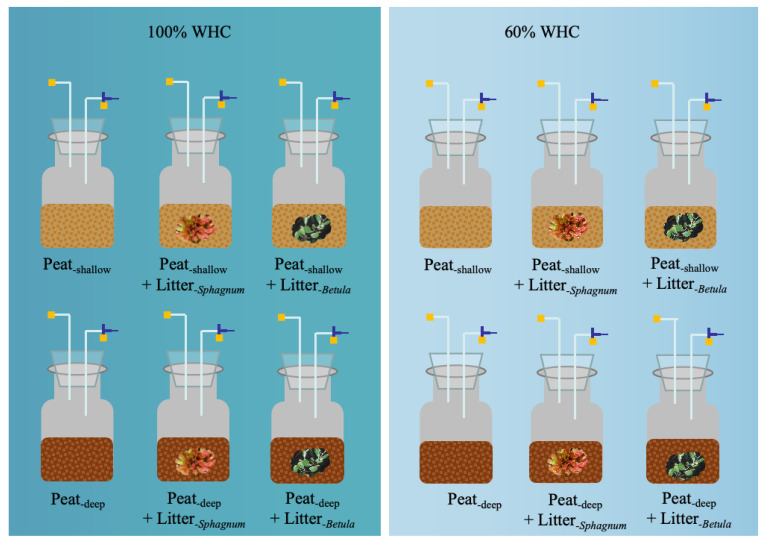
Schematic diagram of experimental design showing 12 combinations (3 litters including control × 2 peats × 2 moistures) of samples incubated at five temperatures. WHC, water-holding capacity.

Peat materials were mixed evenly, and deionized water was added to achieve 100% water-holding capacity (WHC), corresponding to a saturated but not fully waterlogged condition. At this moisture level, the peat was highly saturated and could release water when squeezed, though it was not submerged. Half of the peat at 100% WHC was then air-dried to 60% WHC. The 60% WHC condition represents moisture loss associated with peatland drainage or warming, mimicking drought-like conditions in peatland systems that are normally water-saturated. To maintain consistent headspace, 35 g (fresh weight) of shallow peat and 60 g of deep peat were placed into separate 250 mL glass jars for the incubation experiment. Subsequently, 0.5 g of litter was added into jars and mixed with peat soil. A total of 12 litter, peat, and moisture combinations were incubated at five different temperatures (8, 14, 20, 26, and 32 °C) (Figure 4). In total, 240 samples (3 litters (including control) × 2 depth of peats × 2 moistures × 5 temperatures × 4 replicates) were placed in five growth chambers (PRX-450C, Ningbo Saifu Experimental Instrument Co., Ningbo, China) and incubated in dark with 60% air humidity. During the incubation period, soil moisture was maintained by adding deionized water every two days.

Headspace gas sampling was conducted every other two days starting from day 3 (i.e., on days 3, 6, 9, and 12). During the cultivation period, the glass jars were kept open. At the start of each gas sampling, each jar was sealed with a rubber plug connected to two transparent hoses—one for air extraction and the other for balancing air pressure. Each hose was equipped with one three-way valve. The valves were opened during air extraction and closed afterward. We used four 60 mL syringes to collect gas samples at four intervals over three hours of jar closure: immediately after sealing the jar, and then at 1, 2, and 3 h after closure. A total of 10 ml of headspace gas was collected each time for each sample. After gas collection, the CO_2_ concentration was analyzed using gas chromatography (GC system, Agilent 7890B, Santa Clara, CA, USA). CO_2_ fluxes were calculated according to Formula (1):

(1) R=(C×ρ×V×α×β)/M          

where *R* is the soil respiration rate (μg g^−1^ h^−1^), C is the change in CO_2_ concentration over time during the 4 h of sampling (ppm h^−1^), ρ represents the gas density (mg m^−3^), *V* is the volume of the culture jars (m^3^), *M* is the dry weight of the soil in culture jars, α is the CO_2_ gas mass conversion coefficient, and *β* is the time conversion coefficient.

The sensitivity of CO_2_ release to temperature change is represented by *Q*_10_ and was calculated according to Formulas (2) and (3):

(2) $$R=a{\;\times \;exp}^{b\times T}$$

(3) $$Q_{10}={exp}^{10\times b}\;$$

where *R* is the soil respiration rate at a given incubation temperature, *a* and *b* are the fitting parameters, and *T* is the incubation temperature.

### 4.4. Soil Physicochemical Properties Analyses

Before the incubation experiment, the total nitrogen (TN), total carbon (TC), dissolved organic carbon (DOC), and microbial biomass carbon (MBC) of the shallow and deep peat samples were measured. The TN and TC contents were analyzed using an elemental analyzer (Euro Vector 3000, EuroVector S.p.A., Pavia, Italy), after the dried peat was homogenized using a ball mill (Retsch MM301, Haan, Germany) and weighed into tin capsules. The values were expressed on a dry weight basis. The DOC concentrations were measured using a TOC analyzer (TOC-LCPH/TN, Shimadzu Inc., Kyoto, Japan). For this analysis, 2 g of fresh peat was added to a 50 mL centrifuge tube with 40 mL ultrapure water. The mixture was shaken on a table concentrator at room temperature for 5 h, centrifuged at 4000 rpm for 10 min, and the supernatant was filtered through a 0.4 μm microfiltration membrane using an oil-free diaphragm vacuum pump. Filtrates were stored at 4 °C and measured within 24 h to avoid degradation. Microbial biomass carbon (MBC) was determined using the chloroform fumigation extraction method [75]. Briefly, 10 g of fresh peat was fumigated with ethanol-free chloroform in a desiccator for 24 h at 25 °C. After fumigation, the sample was extracted with 0.5 mol/L K_2_SO_4_ (peat-to-extractant ratio of 1:4, weight/volume) and shaken for 30 min. A parallel set of non-fumigated samples was extracted using the same procedure. The extracts were filtered through Whatman No. 42 filter paper, and the dissolved organic carbon (DOC) was measured using a TOC analyzer (TOC-LCPH/TN, Shimadzu Inc., Kyoto, Japan). The MBC was calculated as the difference in DOC between fumigated and non-fumigated samples, divided by a conversion factor of 0.45. After the incubation experiment, the TN, TC, DOC, and MBC of the peat samples were measured again. The microbial metabolic quotient (MMQ) was calculated by the ratio of microbial respiration to biomass.

### 4.5. Statistical Analyses

Before analysis, the data normality and homogeneity of variance were analyzed, and log-transformation was conducted when necessary. A t-test was used to determine the initial differences in the TC, TN, C:N ratio, DOC, and MBC between shallow and deep peat before the incubation experiment. A multivariate ANOVA was carried out to evaluate the effects of depth, temperature, litter, moisture content, and their interactions on the CO_2_ production rate and DOC concentration. Another one-way ANOVA was employed to assess the effects of litter (control, *Sphagnum*, and *Betula*), soil moisture content (60% WHC and 100% WHC) and depth (shallow and deep peat) on the temperature sensitivity (*Q*_10_) of peat decomposition. Statistical significance was accepted at *p* ≤ 0.05. All the statistical analyses above were performed using *R* software v4.0.3 [76].

## 5. Conclusions

We employed a comparative experiment with two depths of peat under varying moisture content and litter addition to assess peat vulnerability to drought and vegetation change under climate warming. Our study indicates that all of drought, shrub encroachment, and warming can enhance peat decomposition; deep peat exhibits higher temperature sensitivity (*Q*_10_) than shallow peat; and *Sphagnum* litter addition may decrease the microbial metabolic quotient, especially in deep peat. We conclude that, under current and future global warming scenarios, a shift from *Sphagnum* mosses to deciduous shrubs, coupled with drought, will lead to increased peat decomposition and temperature sensitivity, resulting in considerable C loss. Conversely, our findings highlight that restoring *Sphagnum* vegetation and raising the water level could efficiently enhance the C-sink function of degraded peatlands in a warming climate.

## Figures and Tables

**Table 1:** Differences in total carbon (TC), total nitrogen (TN), C:N ratio, dissolved organic carbon (DOC), and microbial biomass carbon (MBC) between shallow (8–13 cm below hummock surface) and deep (35–40 cm below hummock surface) peat (mean ± SE, *n* = 4).

Soil Parameters	Shallow Peat	Deep Peat	*p*
TC (%)	42.02 ± 0.54	30.81 ± 1.89	<0.001
TN (%)	0.91 ± 0.03	1.44 ± 0.08	<0.001
C:N ratio	46.22 ± 1.27	21.78 ± 3.46	<0.001
DOC (mg kg^−1^)	108.53 ± 3.32	176.94 ± 12.29	0.007
MBC (mg kg^−1^)	63.29 ± 13.85	63.53 ± 9.28	0.989

**Table 2:** Effects of depth, temperature, litter addition, drought (moisture content decrease), and their interactions on the CO_2_ production rate (multivariate ANOVA, *p* < 0.05).

Treatments	${\mathrm{CO}}_{2}\;\mathrm{Production}\;\mathrm{Rate}$ (μg g^−1^ h^−1^)		
	df	F	*p*
Depth	1	1395.50	<0.001
Temperature	4	561.90	<0.001
Litter	2	243.88	<0.001
Drought	1	8.51	<0.001
Depth × Temperature	4	80.99	<0.001
Depth × Litter	2	93.97	<0.001
Depth × Drought	1	20.42	<0.001
Temperature × Litter	8	27.59	<0.001
Temperature × Drought	4	3.63	<0.007
Litter × Drought	2	6.61	<0.002
OM × Temperature × Litter	8	14.73	<0.001
OM × Temperature × Drought	4	1.59	0.179
Depth × Litter × Drought	2	0.15	0.865
Temperature × Litter × Drought	8	14.08	<0.001
Depth × Temperature × Litter × Drought	8	4.00	<0.001

**Table 3:** The temperature sensitivity (*Q*_10_) (mean ± SE, n = 4) of peat at different depths with different litter additions under different water-holding capacity conditions. Different lowercase letters represent significant differences in different depths of peat and litter additions (Tukey’s HSD test, *p* < 0.05).

Treatments	*Q*_10_ (100% WHC)	*Q*_10_ (60% WHC)	*F*	*p*
Peat_-shallow_	1.47 ± 0.09 b	1.78 ± 0.03 b	10.05	0.025
Peat_-shallow_ + Litter_-*Sphagnum*_	1.84 ± 0.04 ab	1.81 ± 0.07 b	0.13	0.730
Peat_-shallow_ + Litter_-*Betula*_	1.83 ± 0.11 ab	1.86 ± 0.03 ab	0.11	0.756
Peat_-deep_	1.76 ± 0.03 ab	2.13 ± 0.11 a	7.82	0.049
Peat_-deep_ + Litter_-*Sphagnum*_	1.67 ± 0.13 ab	1.87 ± 0.07 ab	1.84	0.224
Peat_-deep_ + Litter_-*Betula*_	2.10 ± 0.09 a	2.17 ± 0.01 a	0.08	0.785

## Data Availability

Data will be made available on request.

## Supplementary Materials

The following supporting information can be downloaded at https://www.mdpi.com/article/10.3390/plants14152387/s1: Figure S1: The CO_2_ production rate (μg g^−1^ h^−1^) of peat and fresh litter with different treatments. The percentage represents the proportion of CO_2_ produced by fresh litter to the total CO_2_ emitted from both peat and fresh litter combined. Figure S2. Effects of soil moisture (WHC: 100% vs. 60%) on CO_2_ production rates in shallow and deep peat layers at five incubation temperatures, without litter addition. Values are mean ± SE (n = 4). Figure S3: The probability density function (PDF) of microbial biomass carbon (MBC) (mg kg^−1^) after cultivation. Figure S4. Post-incubation microbial biomass carbon (MBC) under different temperature treatments in shallow and deep peat layers. Figure S5. The total nitrogen (TN) contents of *Betula* litter and *Sphagnum* litter.
