# Supplementary material for: Drought and Shrub Encroachment Accelerate Peatland Carbon Loss Under Climate Warming
Source: Plants (Basel). 2025 Aug 2;14(15):2387. doi: 10.3390/plants14152387 (PMC12349620; doi:10.3390/plants14152387)
Supplement: Supplementary file 1 [file plants-14-02387-s001.zip › plants-3773004-supplementary.pdf]

Supplementary material

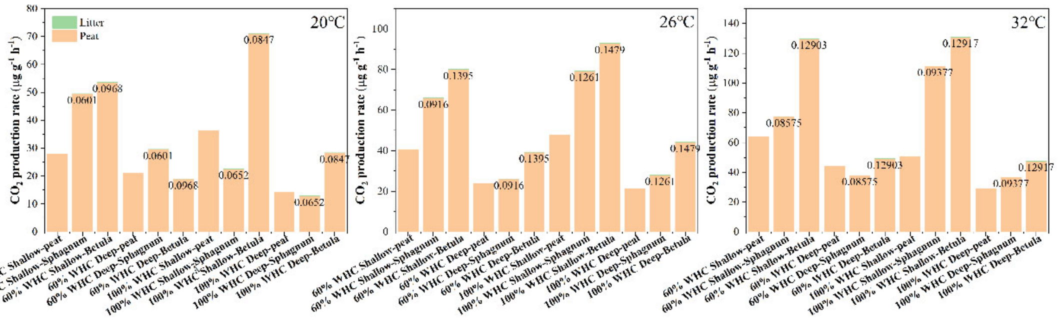

Figure S1. The CO_2 production rate (μg g^{-1} h^{-1}) of peat and fresh litter at different treatments.

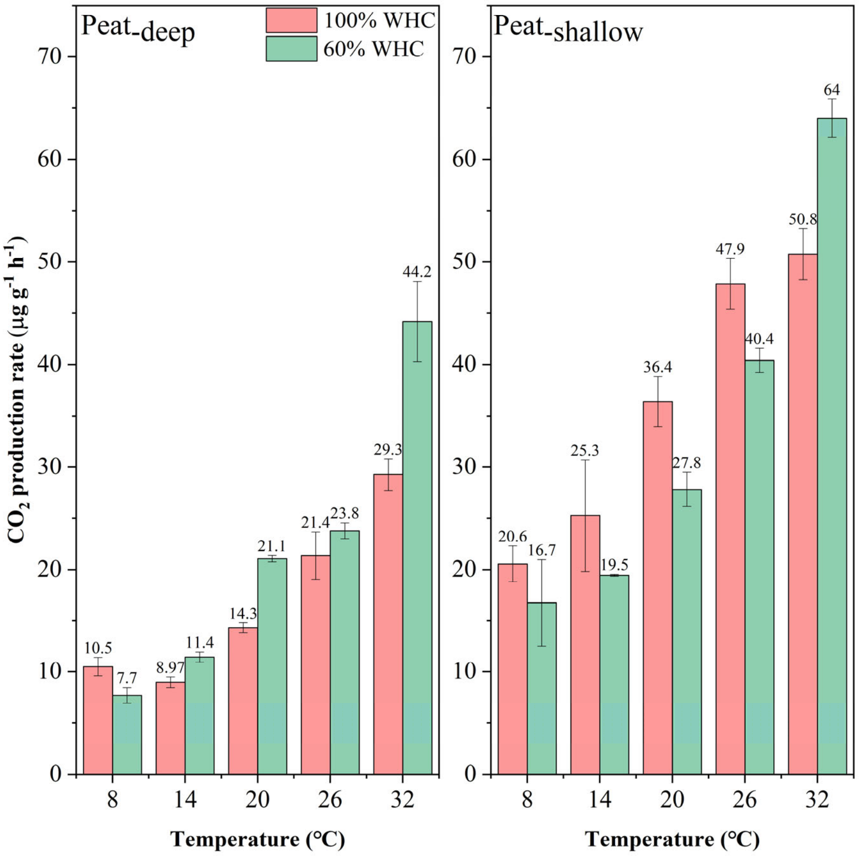

Figure S2. Effects of soil moisture (WHC: 100% vs. 60%) on CO_2 production rates in shallow and deep peat layers at five incubation temperatures, without litter addition. Values are mean ± SE (n = 4).

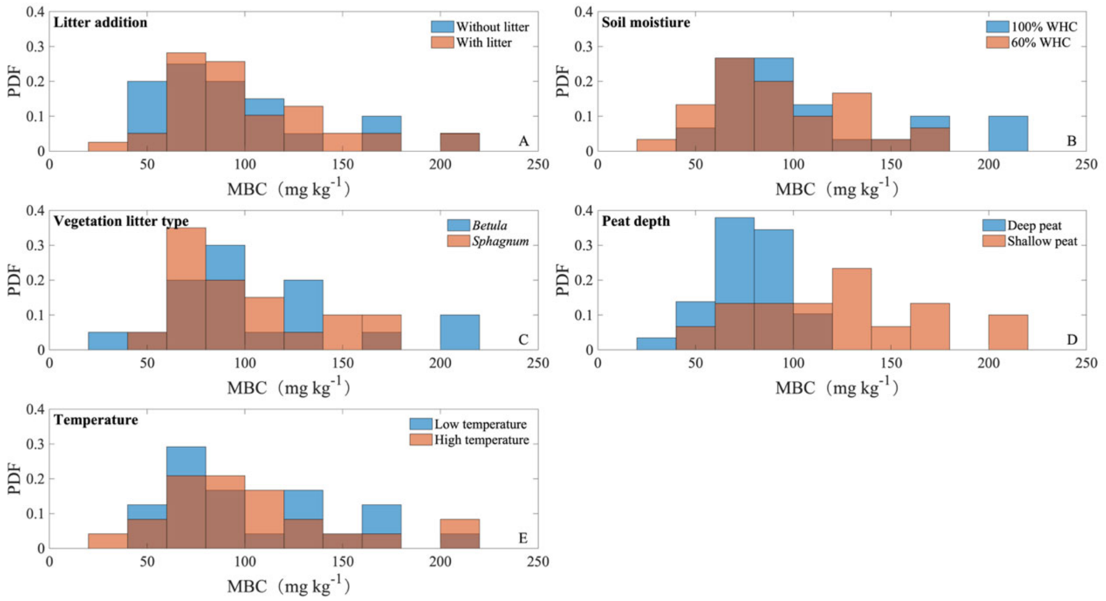

Figure S3. The probability density function (PDF) of microbial biomass carbon (MBC) (mg kg^{-1}) after cultivation.

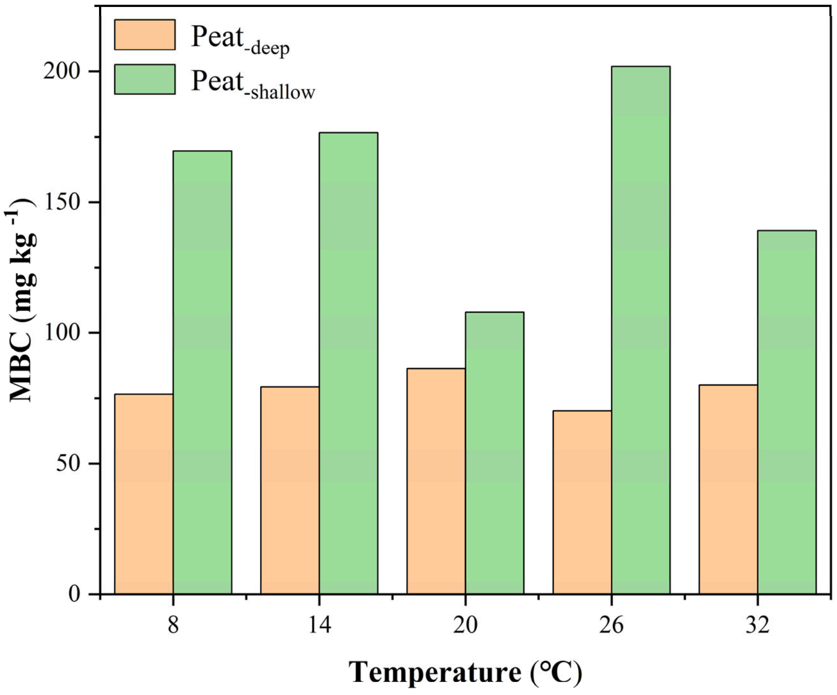

Figure S4. Post-incubation microbial biomass carbon (MBC) under different temperature treatments in shallow and deep peat layers.

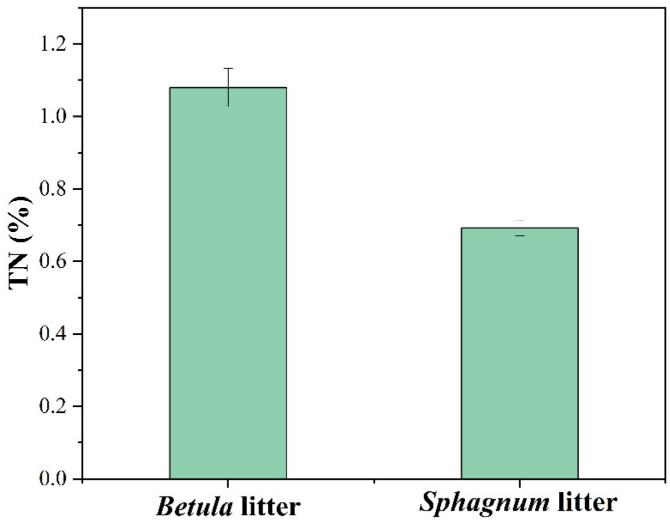

Figure S5. The total nitrogen (TN) contents of *Betula* litter and *Sphagnum* litter.
